# Improving the efficiency of rear emitter silicon solar cell using an optimized n-type silicon oxide front surface field layer

**DOI:** 10.1038/s41598-018-28823-x

**Published:** 2018-07-13

**Authors:** Sangho Kim, Jinjoo Park, Pham Duy Phong, Chonghoon Shin, S. M. Iftiquar, Junsin Yi

**Affiliations:** 1 Department of Energy Science, Sungkyunkwan University, Natural Sciences Campus, 2066 Seobu-ro, Jangan-gu, Suwon-si, Gyeonggi-do 16419 South Korea; 2 College of Information and Communication Engineering, Sungkyunkwan University, Natural Sciences Campus, 2066 Seobu-ro, Jangan-gu, Suwon-si, Gyeonggi-do 16419 South Korea

## Abstract

Optical and electrical characteristics of n-type nano-crystalline-silicon oxide (n-µc-SiO:H) materials can be varied to optimize and improve the performance of a solar cell. In silicon heretojunction (SHJ) solar cells, it can be used to improve carrier selectivity and optical transmission at the front side, both of which are vitally important in device operation. For this purpose, the n-µc-SiO:H was investigated as the front surface field (FSF) layer. During film deposition, an increased CO_2_ flow rate from 0 to 6 sccm resulted in changes of crystalline volume fractions from 57 to 28%, optical band-gaps from 1.98 to 2.21 eV, dark conductivities from 7.29 to 1.1 × 10^−5^ S/cm, and activation energies from 0.019 to 0.29 eV, respectively. In device applications, a minimum optical reflection was estimated for the FSF layer that was fabricated with 4 sccm CO_2_ (FSF-4), and therefore obtained the highest external quantum efficiency, although short circuit current density (J_sc_) was 38.83 mA/cm^2^ and power conversion efficiency (PCE) was 21.64%. However, the highest PCE of 22.34% with J_sc_ = 38.71 mA/cm^2^ was observed with the FSF prepared with 2 sccm CO_2_ (FSF-2), as the combined opto-electronic properties of FSF-2 were better than those of the FSF-4.

## Introduction

The performance of high-efficiency silicon solar cells depend on the passivation of surface defects^1,2^, available light to the absorber layer^3^ and efficient as well as selective collection of photo-generated charge carriers^4,5^. Although there are several other parameters on which the device performance depends, the three stated above are considered as very important. Therefore, many studies have been conducted on these parameters. Our present investigation is with thin-film, wide band-gap, n-type nano-crystalline silicon oxide (n-µc-SiO:H) material and their application as an optimized front surface field (FSF) layer in silicon heretojunction (SHJ) solar cells; where the aim is to make more light available to the absorber layer and improve selectivity in carrier collection. A wide band-gap and a highly doped layer can facilitate sharp band-bending, which in turn facilitates carrier selectivity. For example, molybdenum-oxide and magnesium-oxide can be used as carrier selective contacts^6,7^. Wide band-gap silicon oxide can also be used as a carrier-selective contact layer^5^. One major advantage of thin-film amorphous or nano-crystalline materials is that their optical band-gap, or transparency, can be easily altered by varying deposition conditions. In n-µc-SiO:H, these two parameters follow opposite trends. Therefore, an optimization of the n-µc-SiO:H layer becomes necessary for its suitable application in a SHJ solar cell.

In 2017, one of the highest ever power conversion efficiencies (PCE) for silicon solar cells was reported (26%^8,9^) by the Kaneka corporation, Japan. However, the material and technology adopted to fabricate this device seems to include the expensive inter-digitated back contact (IBC)^10–12^ method. According to the 2018 photovoltaic-report, prepared by the Fraunhofer Institute for Solar Energy System, ISE^13^, Japan remains one of the lowest module-producing countries. It is well known that the cost of producing a solar cell is a crucially important factor. Therefore, a cost-effective heterojunction crystalline silicon or SHJ solar cell with a moderate PCE^14–19^ seems promising for large-scale applications.

One of the advantages of the IBC solar cells is that more light can be made available to the absorber layer, because the doped layer and electrodes are absent at the front surface^8,20^. A higher device efficiency can also be obtained by using light-trapping schemes in other device structures^21–24^, in comparison to that without the light trapping. Fundamentally, the coupling of light into the absorber layer of a solar cell is critically important; higher the intensity of the coupled light, higher is the current density and PCE. However, in a thicker SHJ solar cell the effect of the back reflector may be lesser than that in a thinner cell^25^. Therefore, improved light management at the front side of the SHJ solar cell becomes necessary. Herein, our attempt is to improve light coupling at the front surface of a single-junction SHJ solar cell, at the same time maintain an efficient and selective carrier collection. This approach will be useful not only in the single junction SHJ solar cells but also in a two-terminal tandem device structures, and bifacial solar cells.

The back surface field (BSF) and front surface field (FSF) solar cells are different in the sense that in the BSF device structure, light enters through the emitter while in the latter, the emitter is located at the back of the cell. Furthermore, in many BSF solar cells the back of the device is covered with opaque electrodes, while in the case of FSF devices, the electrode connected to the FSF layer has to be optically transparent to allow the maximum possible light into the absorber layer. In the present investigation, we used a FSF device structure. One of the reasons for choosing this structure is that we can use a wide-optical band-gap and a highly conducting n-µc-SiO:H layer^26^. Furthermore, similar to intrinsic amorphous silicon oxide^27,28^, the optical band-gap, refractive index, and electrical conductivity can suitably be altered in the n-µc-SiO:H layer^26^, opening up a possibility of matching its opto-electronic properties to the two surrounding layers, thereby minimizing opto-electronic loss in the photovoltaic process.

As mentioned earlier, efficient capture of the incident light at the front surface and its transmission into the absorber layer of a solar cell is necessary to improve its photovoltaic conversion efficiency. In this context, a textured front surface can also reduce the surface reflection of incident light^22–24,29^; in such a surface, multiple optical reflections of the incident light rays may take place, thereby reducing its overall optical reflectivity. Light that enters the textured front surface can undergo further reflection loss at the interfaces of different layers. In a BSF solar cell, p-type amorphous silicon (p-a-Si:H) is popularly used as an emitter (with an n-type c-Si absorber material)^16,17^. The p-a-Si:H layer is known to exhibit higher optical absorption than n-µc-SiO:H^26,30–32^. At a similar optical band-gap, n-µc-SiO:H shows better electrical conductivity than the p-type emitter.

**Figure 1:**
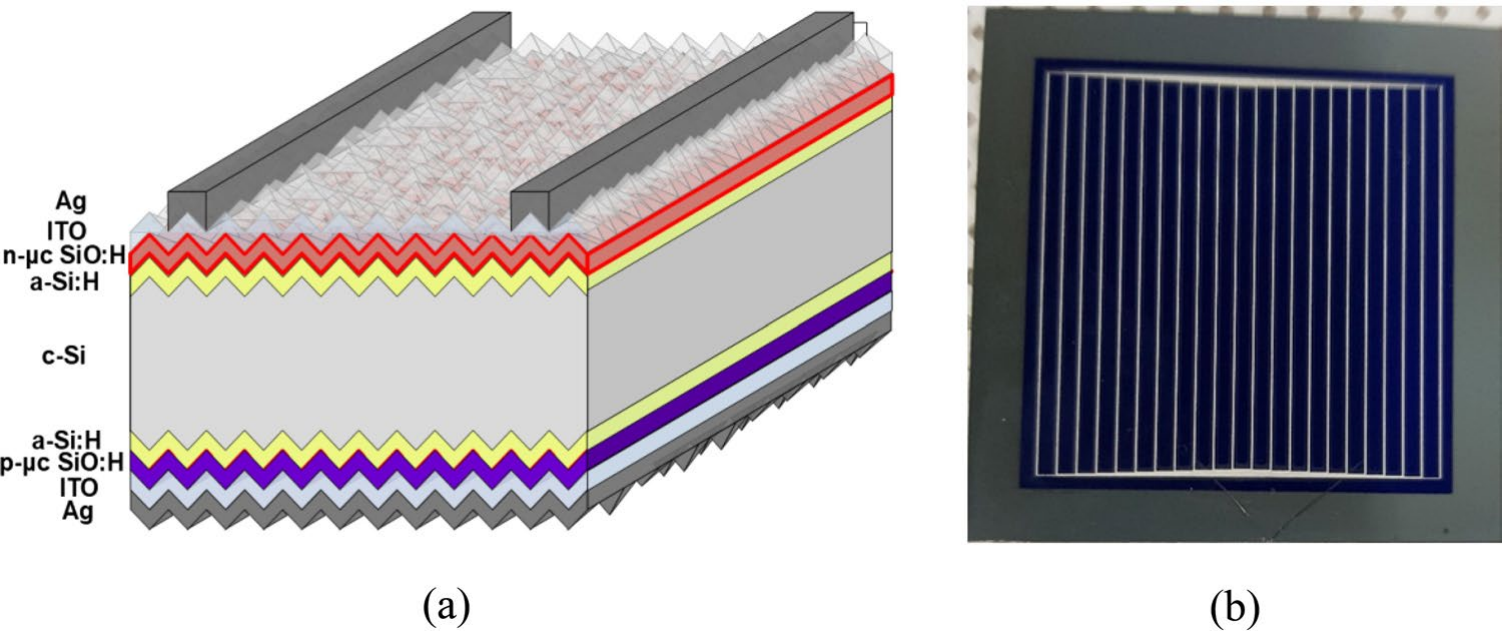
(**a**) Schematic diagram of the layer structure of solar cells. Thicknesses of the ITO front and back layers are 80 and 120 nm, respectively. (**b**) Top-view image of the front side of a 32 mm × 32 mm solar cell.

Solar cells contain multiple thin layers. For example, at the front side of our device, as shown in Fig. 1(a), an indium tin oxide (ITO) electrode, n-µc-SiO:H as the FSF, and a passivation layer of intrinsic a-Si:H are present. The differences in the refractive indices of two adjacent layers can lead to light being partially reflected. This reflection can be described by Fresnel’s relation. At the ITO/n-µc-SiO:H and n-µc-SiO:H/a-Si:H interfaces, these reflections contribute to a loss of light that we want to reduce. An optimum refractive index (RI) of the n-µc-SiO:H layer is expected to reduce such reflection losses. Furthermore, carrier selectivity of the FSF layer is also important. The n-µc-SiO:H layer can exhibit a wide range of opto-electronic properties. Therefore, we investigated the thin film n-µc-SiO:H as a front surface field (FSF) window layer, in order to improve the device performance.

## Experimental Details

Solar cells were fabricated using commercially available Czochralski-grown n-type c-Si wafers (resistivity 1–10 Ω.cm, thickness 200 µm, <100> oriented) as the absorber material. Both surfaces of the wafers were pyramidally textured using an anisotropic wet-etching method in a dilute alkaline solution^33^. The wafers were then sequentially cleaned with acetone, methanol, de-ionized water, RCAl, and RCA2. Immediately before depositing the intrinsic amorphous silicon (a-Si:H) passivation layer, native oxides on the surfaces of the wafers were removed by dipping into l% hydrofluoric acid for one minute. Intrinsic a-Si:H^34^ and doped silicon oxide layers^26,35^ were deposited using a cluster-type multi chamber plasma-enhanced chemical vapor deposition (PECVD) system. A schematic diagram of the layer structure of the solar cells is shown in Fig. 1(a) and an image of the top view of the solar cell is shown in Fig. 1(b). Table 1 shows the deposition conditions of the thin-film silicon alloy materials. The a-Si:H passivation layer and n-µc-SiO:H as FSF layer were deposited on one side of the wafers (front side of the solar cell). On the other side, the a-Si:H passivation and p-µc-SiO:H emitter layers were deposited; which makes them rear-emitter solar cells. Silane (SiH_4_) and hydrogen (H_2_) source gases were used while preparing the PECVD layers. In addition to these source gases, carbon dioxide (CO_2_) and phosphine (PH_3_) were used to prepare the n-µc-SiO:H layer, while CO_2_ and diborane (B_2_H_6_) were used for the emitter layer. For convenience, we refer to the films prepared with CO_2_ flow rates of 0, 2, 4, and 6 sccm as Film-A, -B, -C, and -D respectively, while the corresponding cells are denoted as Cell-A, -B, -C, and -D respectively. Here, Film-A and FSF of Cell-A were n-type nano-crystalline silicon (n-μc-Si:H), without oxygen, while the other films and FSF layers had oxygen. ITO layers were sputter deposited^17^ on the emitter and FSF layers. On the front side of the solar cells, silver electrodes were formed by screen printing with an Ag-paste^33,36^, as shown in Fig. 1(a,b). The height and width of the Ag electrodes were 20 µm and 40 µm respectively. The back of the solar cell was also screen printed with the Ag paste, but covering the whole surface area of the solar cell. The printed Ag paste was dried by firing at a temperature of l60 °C in a furnace. The total surface area of the rectangular-shaped solar cell is 10.24 cm^2^. Suitable metal masks were used in all the depositions steps. The external quantum efficiency (EQE) of the SHJ solar cells was evaluated by using an EQE measuring system (supplied by PV measurement Inc., QEX7). The performance of the solar cells was characterized and estimated using the current-voltage (I-V) characteristic curves obtained under Air Mass 1.5 Global insolation (100 mW/cm^2^, AMl.5 G) at 25 °C.

**Table 1:** RF PECVD deposition conditions of various silicon alloy layers.

Materials	Thickness (nm)	PECVD Electrode Distance (mm)	T_s_ (°C)	Power Density (mW/cm^2^)	Pressure (Torr)	SiH_4_ (sccm)	H_2_ (sccm)	B_2_H_6_ (sccm)	PH_3_ (sccm)	CO_2_ (sccm)
i-a-Si:H	7	80	180	128	0.8	30	150	0	0	0
p-μc-SiO:H	30	20	100	43	1.5	8	900	0.008	0	2
n-μc-SiO:H	30	20	100	24	1.5	4	500	0	0.08	0, 2, 4, 6

The i-a-Si:H layers are passivation layers, p-μc-SiO:H as emitter and n-uc-SiO:H as front surface field (FSF) layers, here T_s_ is substrate temperature.

A variation in the oxygen content of intrinsic or doped silicon oxide materials^26–28,30,35^ can alter their optical absorption, optical band-gap and refractive index. Therefore, we varied the oxygen content in the n-µc-SiO:H layer by varying the CO_2_ flow rate during film deposition, so that more light can be coupled into the solar cell, thereby enhancing electrical output from the device.

The thicknesses and optical constants of the films were measured using spectroscopic ellipsometry (VASE, J.A. Woollam, 240 nm < λ < 1700 nm) at room temperature, and by using the Cody-Lorentz method combined with the Bruggemen effective medium approximation (BEMA) model^37^. The electrical conductivity of the samples was measured using a coplanar electrode configuration on the films that were separately deposited on glass substrates. We used a programmable Keithley 617 electrometer for conductivity measurements. However, as the electrical conductivity in a solar cell is not exactly of planar type but it travels normal to the plane of (or perpendicular to) the interfaces of the silicon alloy materials. Therefore, the perpendicular electrical conductivities were measured by conductive atomic force microscopy (C-AFM) (Model PSIA, XE150). The C-AFM results report the distribution of electrical conductivity across the surface area of the films. C-AFM measurements were performed with a conventional AFM system, but with an additional measurement of electrical current between the sample mounting platform and AFM probe tip. For this measurement, the films were deposited on crystalline silicon wafers.

Theoretically, we estimated optical reflection at an interface, by using Fresnel’s relation. Energy band diagrams were drawn using AFORS-HET simulation program^38^. We used the numerical values of measured optical band-gap, doping concentration, approximately 3.9 eV as electron affinity. Furthermore, we simulated device characteristics, when the characteristics were similar to the experimentally measured one; we use those conditions for estimating the band diagrams. The diode equivalent parameters of the cells were estimated by using single diode model, and by numerical simulation^39,40^.

## Results and Discussion

### Optoelectronic and structural properties of n-µc-SiO:H FSF layers

**Figure 2:**
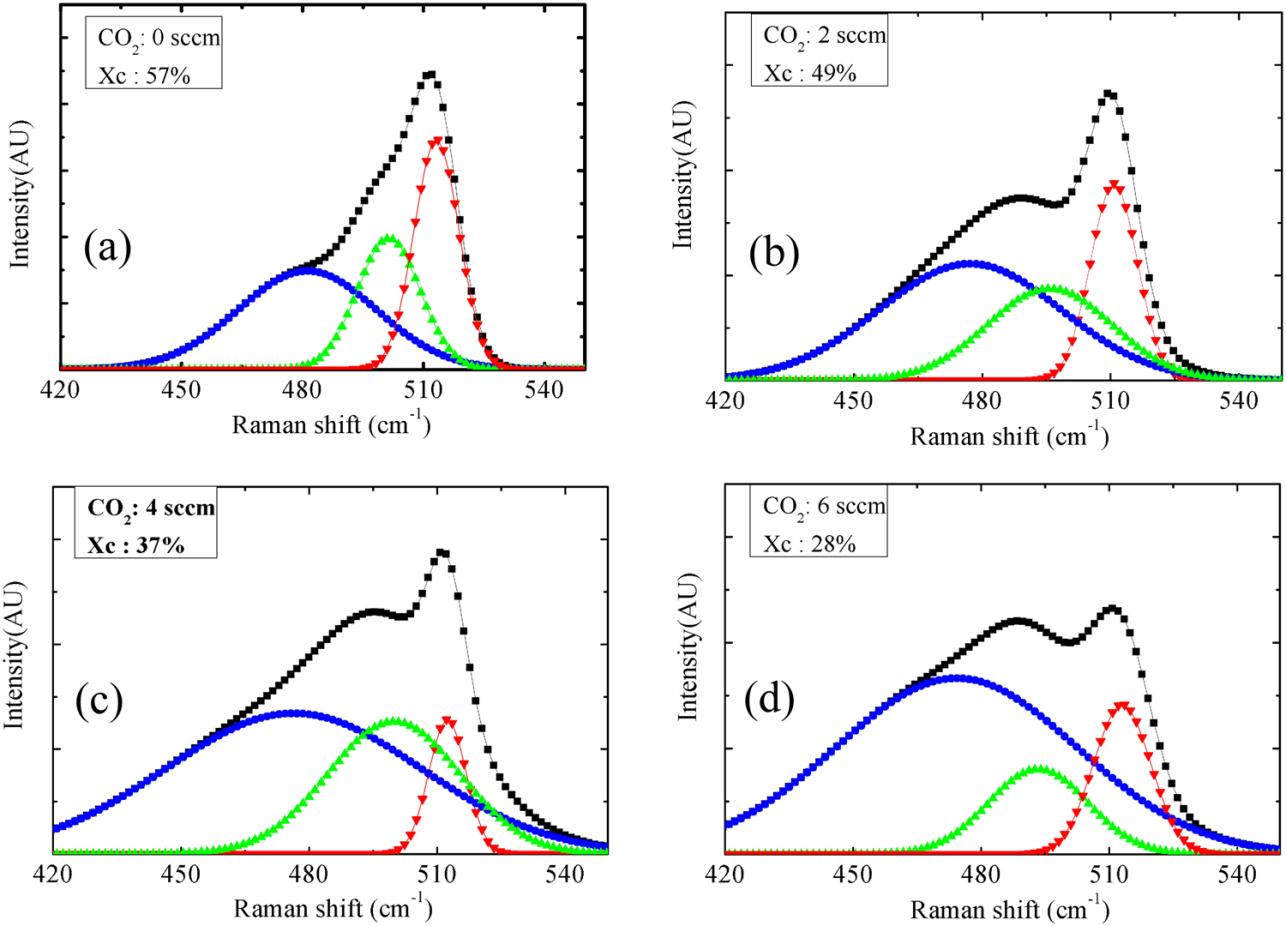
Raman spectra of the n-µc-SiO:H films deposited at CO_2_ flow rates of (**a**) 0, (**b**) 2, (**c**) 4, and (**d**) 6 sccm.

Our investigation is primarily concerned with the variation in the optoelectronic properties of the n-µc-SiO:H layer, therefore, the CO_2_ flow rate was altered to prepare different n-type layers. Investigation on the individual n-µc-SiO:H layers and their application in solar cells was conducted separately. In order to analyze their optoelectronic and structural properties, n-µc-SiO:H films were deposited on glass substrates. We used Raman spectral analysis to estimate the crystalline volume fraction (X_c_); the following relation (1) was used to estimate X_c_.

1 $${{\rm{X}}}_{{\rm{c}}}=({{\rm{I}}}_{510}+{{\rm{I}}}_{500})/({{\rm{I}}}_{510}+{{\rm{I}}}_{500}+{{\rm{I}}}_{480})$$

where I_510_, I_500_, and I_480_ are the intensities of the deconvoluted peaks of Raman spectra at around 510 cm^−1^, 500 cm^−1^ and 480 cm^−1^, respectively. The peak centered at 480 cm^−1^ is considered to be related to amorphous phase. The Raman spectra of the four different films are shown in Fig. 2, of which three films contain n-µc-SiO:H (Fig. 2(b–d)) and one contains n-µc-Si:H (Fig. 2(a)). Here, the black curves represent experimentally measured data points, while the plots of other colors represent deconvoluted Gaussian peaks. The results show that with an increase in the CO_2_ flow rate, the nano-crystallinity within the films decreased. The peak near 510 cm^−1^ is related to nano-crystalline silicon (µc-Si:H), while the other peaks (~480 and 510 cm^−1^) are due to various phases amorphous silicon alloy. The peaks corresponding to I_510_, I_480_ in Fig. 2(a) are attributed to the amorphous silicon material, while the peaks in Fig. 2(b–d), contain information on both n-type amorphous silicon (n-a-Si:H) and n-type amorphous silicon oxide (n-a-SiO:H). This trend in the Raman spectra shown in Fig. 2(b–d), can be attributed to the two-phase structure of the silicon oxide material^41^; a silicon-rich phase and an oxygen-rich phase. When the volume fraction of the oxygen-rich phase increases, the volume fraction of the silicon-rich phase decreases. Such a decrease in the silicon-rich phase primarily indicates a decrease in the nano-crystalline silicon phase. Therefore, the X_c_ can be considered proportional to the volume fraction of the highly conducting silicon-rich phase.

**Figure 3:**
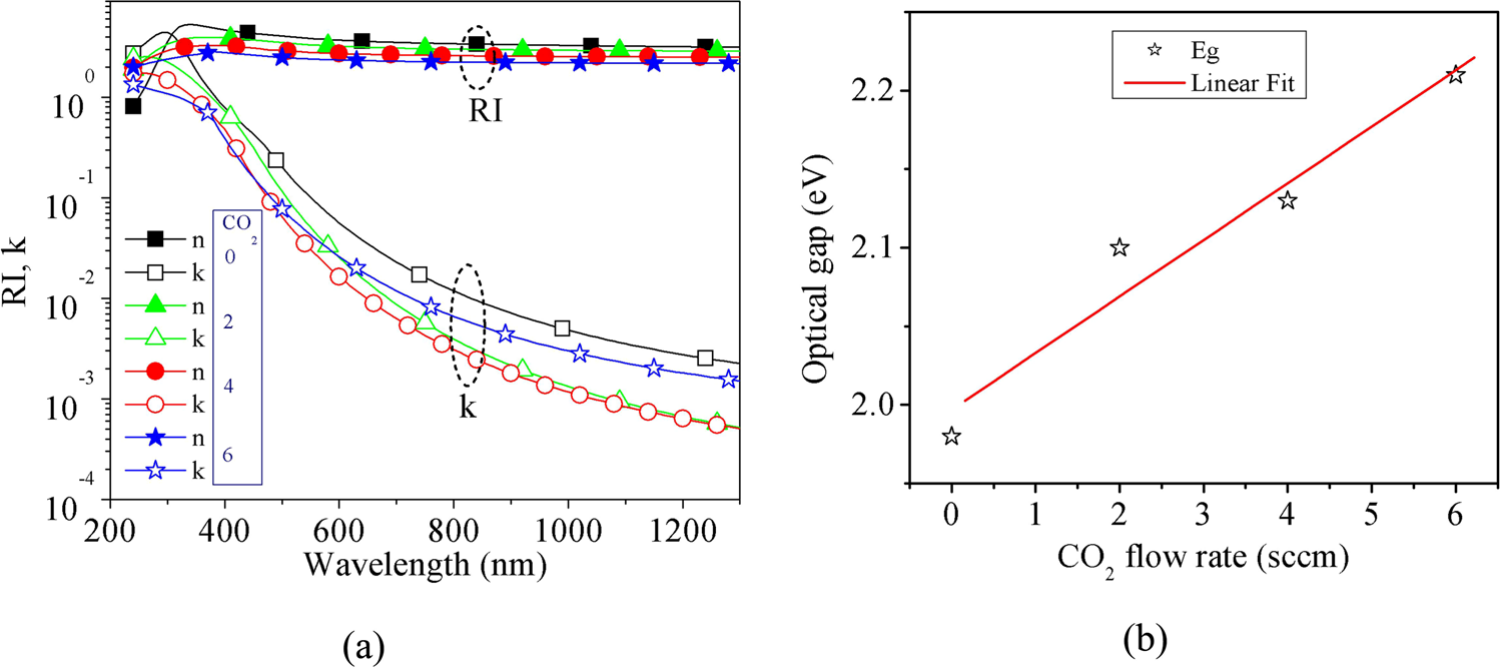
(**a**) Variation in the RI and k of different FSF films. Fewer point markers are shown here. The continuous lines join the data points. (**b**) Optical band-gap, estimated using the Tauc plot, is shown using star markers. The straight line is a linear fit to the data points.

Figure 3(a) shows the wavelength dependant refractive index (RI) and extinction coefficient (k) of n-µc-SiO:H films, which were estimated from spectroscopic ellipsometric measurements. These data were used to estimate the optical absorption coefficient by using $\alpha =\frac{4\pi k}{\lambda }$. The optical band-gap (E_g_) of the films were estimated with the help of Tauc plot following the equation, $\sqrt{\alpha h\nu }=B(h\nu -\,{E}_{g})\,$, where λ is wavelength, ν is frequency, *h* is Planck’s constant, and B is another constant. The RI was used to estimate optical reflection at the interfaces.

The optical-gaps of the films are shown in Fig. 3(b) with star symbols, while the line is a linear fit to the data points. This indicates that the optical band-gap increases almost linearly with the CO_2_ flow rate. Comparing the variation in E_g_ to the trend of variation in intrinsic silicon oxide films^27^, it can be stated that the linear variation in E_g_ may be an oversimplification; however, we consider this linear trend to be reasonable within the small range. This trend of variation is similar to the previously reported results; the electrical conductivity of silicon oxide films decreases with an increase in oxygen content within the film or with an increase in the CO_2_ flow rate during its deposition^26–28,30,31,35^, as was observed for intrinsic^27,28,42^, p-type^30^ as well as n-type n-µc-SiO:H films^26^.

**Figure 4:**
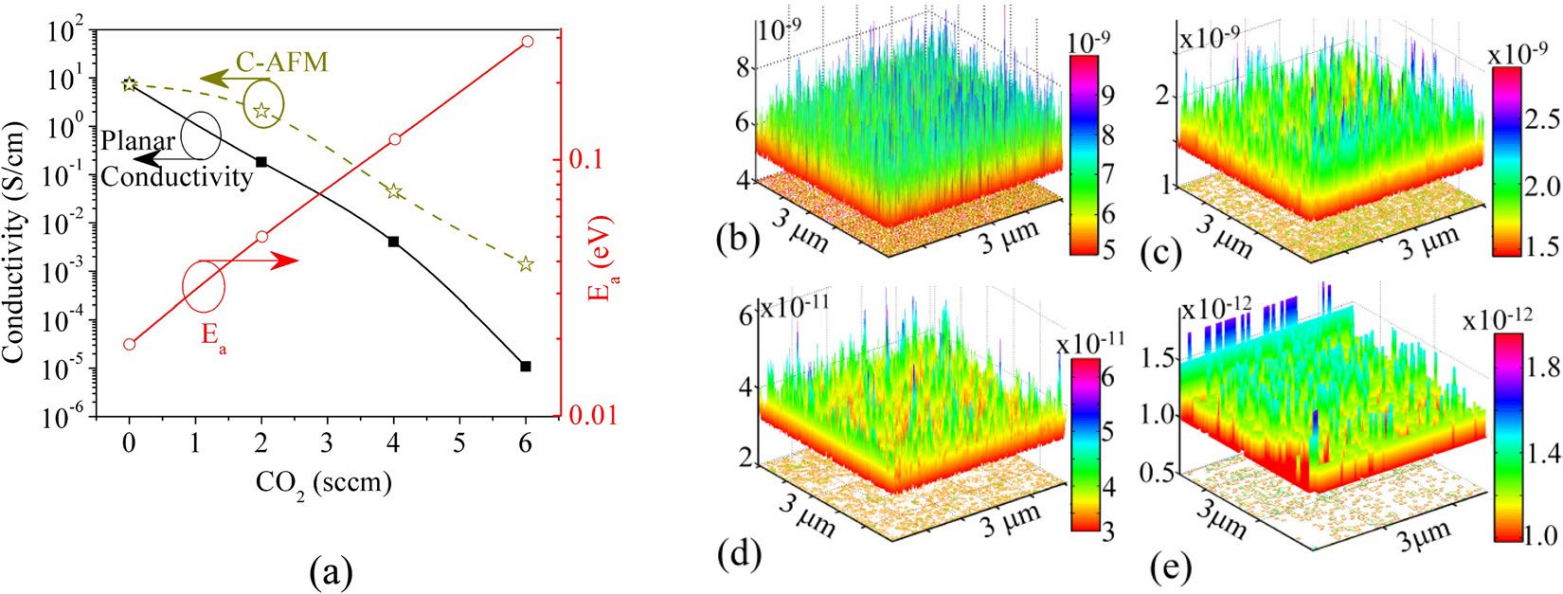
(**a**) Variation in the planar conductivity, C-AFM conductivity, and E_a_ with CO_2_ flow. (**b**–**e**) Show the C-AFM currents of n-µc-SiO:H films. Taking n-µc-Si:H as the reference, the AFM-conductivity is shown in (**a**) with star markers and labeled as “C-AFM.”

When the crystalline volume fraction in a film changes, the number density and size of the crystallites are also expected to change. Nano-crystalline silicon has a higher electrical conductivity than amorphous silicon or amorphous silicon oxide. We observed such a variation in the electrical conductivity by measuring the electrical (dark) conductivity in a planar electrode configuration (planar-conductivity), where current flows along the plane of the film, as well as in a direction perpendicular to the plane of the film (AFM-conductivity or C-AFM). The relationship between planar-conductivity and its activation energy (E_a_), with the CO_2_ flow rate, is nearly exponential, as shown in Fig. 4(a). Figure 4(b–e) shows the current measured by C-AFM. The C-AFM probe scans over a 3 × 3 µm^2^ surface area. The Film-A exhibits the highest average C-AFM-current, while Film-D exhibits the lowest. Generally, under identical current-measuring conditions the measured current is proportional to electrical conductivity. If we assume that the planar- and AFM-conductivities are the same for Film-A, then the variation in planar- and AFM-conductivity will look like as shown in Fig. 4(a) with yellow-colored star markers. In other words, the AFM conductivities were normalized with the planar conductivity of the Film-A. It shows that both types of electrical conductivity decrease from Film-A to Film-D. In the case of Film-A the observed maximum C-AFM-current was as high as 500 pA, while that of Film-B decreased to ~100 pA; in the rest of the films a continuous decreasing trend was observed. The planar distribution of local electrical currents in C-AFM measurements shows that current varies from one point to another. This variation may be ascribed to the local distribution of silicon crystallites on the surface; when the C-AFM probe tip touches the silicon crystallites, a high current was observed, while in the amorphous phase the C-AFM current was lower. Therefore, we estimated the C-AFM conductivity by calculating the average probe current. By comparing Figs 3(b) and 4(a), we can infer that although an increase in the optical band-gap of the films indicates a high optical transparency, it is associated with a decreased electrical conductivity. Higher conductivity, especially the C-AFM conductivity, will help in better carrier transport, thereby a reduced recombination loss can be expected. The C-AFM conductivity is higher for Film-B than the Film-C and Film-D. Therefore, a better electron transport is expected across the FSF-2 layer (prepared with 2 sccm of CO_2_) than the others. As a result, a lower recombination of carriers around the FSF is expected with this film.

### Light reflection due to the n-µc-SiO:H FSF layer

**Figure 5:**
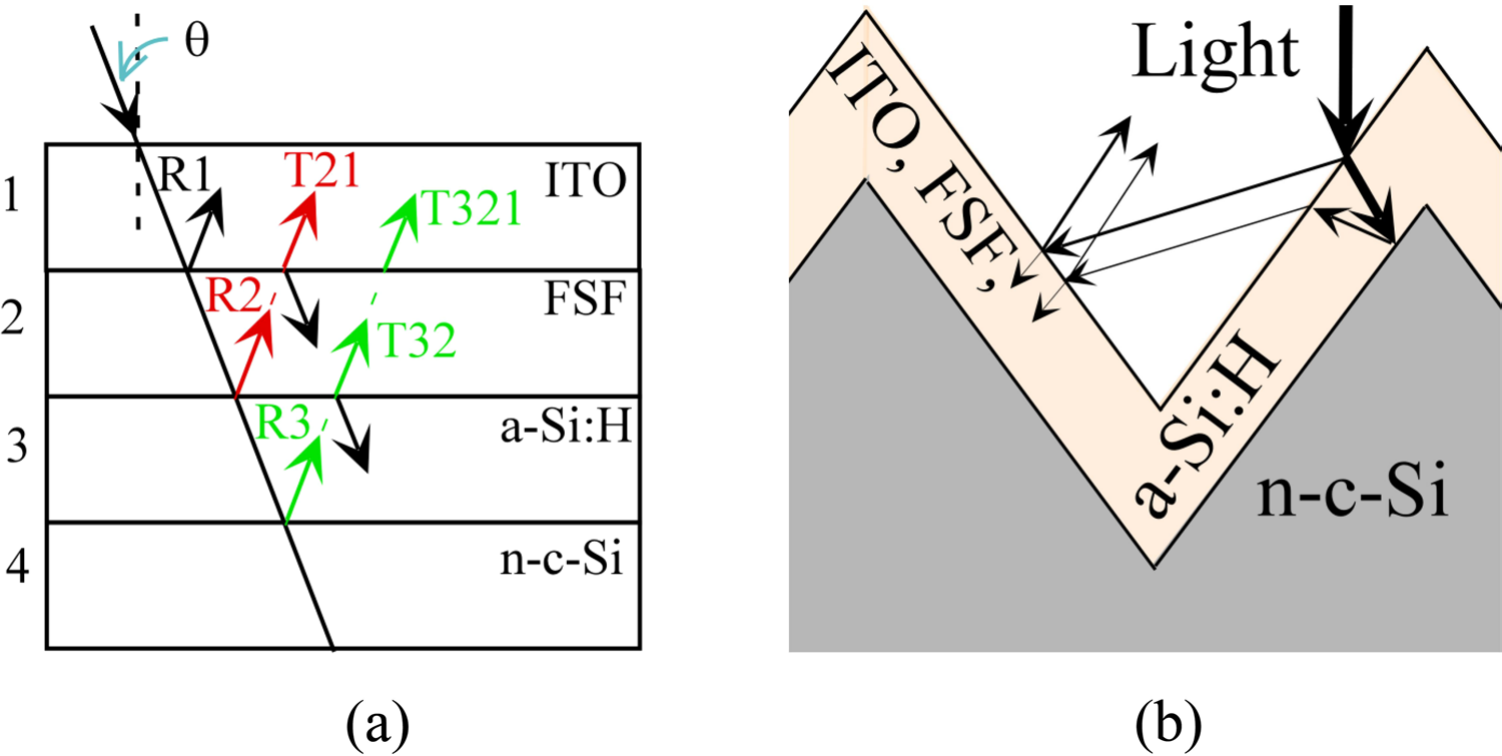
(**a**) Schematic diagram of the layer structure used in estimating the reflection of light. The incident ray and reflections/transmission are indicated by arrows. θ is the angle of incidence, which is equal to the angle of transmission at normal incidence. (**b**) A simplified schematic and optical ray diagram of the textured front surface of the solar cells.

In addition to the variation in the electrical conductivity of n-µc-SiO:H films, as shown in Fig. 4, their optical properties also change, as shown in Fig. 3. A variation in the optical (RI and k) parameters will have an impact on the light transmitted through the n-µc-SiO:H layer. We assume that the 30 nm thick FSF layer and 7 nm thick passivation layers have a very small or negligible optical absorption. Even as the layers will have some optical absorption, yet its variation from one FSF layer to another will be further smaller, therefore, we neglected optical; absorption for estimating the reflection. However, a variation in RI can alter light reflection at the interfaces. This reflection was estimated by using the RI data in Fig. 3(a) and following Fresnel’s relation. Various interfaces and light rays considered in the estimation are shown in Fig. 5(a). On the front side, the reflection should be low so that more light can enter the absorber layer, while at the back side of the solar cell, a higher optical reflection can help in light trapping; as it can send the unabsorbed light back to the absorber layer for further absorption. Here, one of our primary interests is to reduce optical reflection at the front surface by optimizing the n-µc-SiO:H FSF layer. Generally, the RI of silicon oxide films can be changed by altering their oxygen content^26–28^. This can be achieved by a controlled variation in the CO_2_ flow rate during film deposition. We selected four different silicon oxide materials, Film-A, B, C, and D, as we found that these layers have good conductivity and optical transparency. In order to reduce the reflection loss, the RI of the silicon oxide layer should be optimized. At first, we used flat surfaces for the theoretical estimation, as it is easier and more accurate to calculate than that of a textured surface. Furthermore, a simple relation may exist between the reflectivity of flat and textured surfaces^24,29^ and the trends of variation in the reflectivity of flat or textured surfaces are expected to be similar. Therefore, we investigated reflection from flat surface, as indicated in schematic diagram in Fig. 5(a). Figure 5(b) shows a simplified schematic diagram of the ITO, FSF, and a-Si:H on textured n-c-Si surfaces.

**Figure 6:**
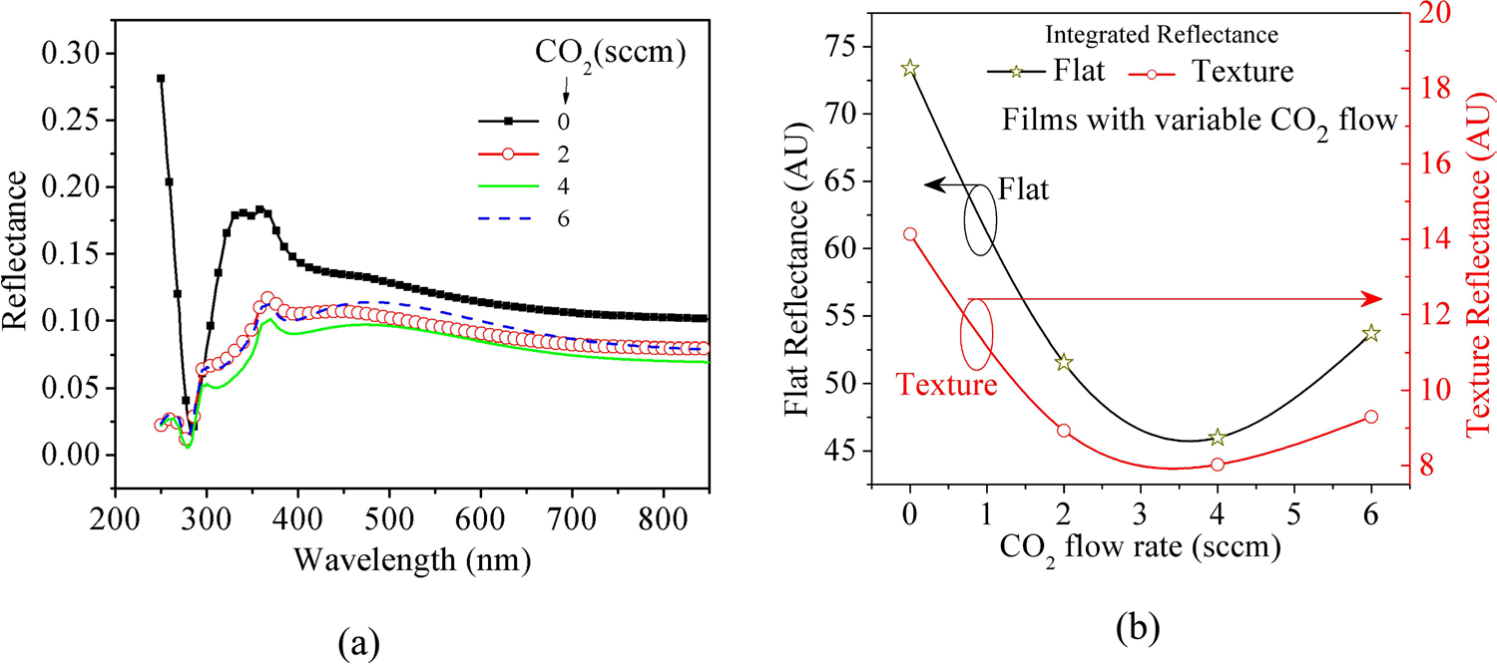
Theoretically estimated total optical reflection at the flat front surface of the solar cells, when θ = 0°. (**a**) Spectral distribution of reflectance and (**b**) integrated reflectance for the flat surface at θ = 0° (open ‘star’ symbol) and textured surface at θ = 50° (open ‘circle’ symbol).

Reflectance at ITO/FSF and FSF/a-Si:H interfaces were estimated because optical reflection at these two surfaces varies due to a variation in the FSF layer. We neglect the optical interference because the films are very thin. Figure 6(a) shows that the Film-A exhibited the highest spectral reflection. The lowest reflection was observed for Film-C. The reduction in reflection loss can be observed over the entire spectral range in Fig. 6(a). To obtain a clearer understanding of the trend of overall reflection, we integrated the reflectance, as shown in Fig. 6(b). In Fig. 6(b) we observe that a minimum exists for Film-C. So it is expected that, in our device structure, the FSF fabricated with 4 sccm CO_2_ will result in the lowest reflection. Here, we estimated the optical reflection at θ = 0° for the flat interfaces. While the reflectance for the textured surfaces were estimated with θ = 50°, this estimation is approximated by equating the angle of incidence and angle of refraction to 50° and neglecting its variation with wavelength.

### Solar cell device characteristics

**Figure 7:**
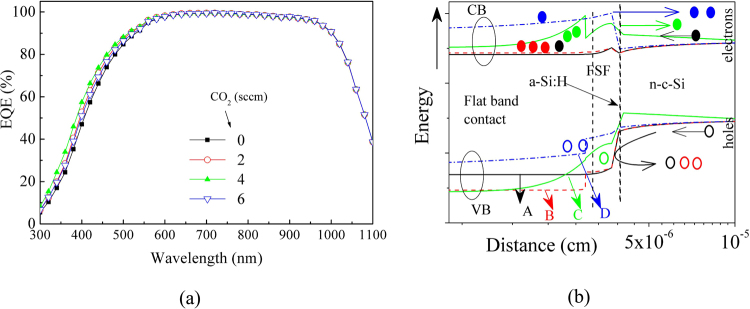
(**a**) EQE spectra of the solar cells under AM1.5 G illumination. (**b**) Schematic energy-band diagram of the cells, estimated using the AFORS-HET simulation program^38^ under steady-state conditions. Most of the layer properties are taken from Figs 3 and 4.

On the basis of the above results, we expect that even if the variation in the optical absorption of the FSF layer is negligible, the variation in light reflection at its front and back surfaces will have some influence on the device performance. If the optical reflection is reduced or optimized along with its electrical characteristics, we can achieve a better device performance. The measured EQE spectra of the solar cells with these layers, as shown in Fig. 7(a), can be used to analyze this aspect. A variation in the blue (or short wavelength) response of the EQE from Cell-A to Cell-D can be clearly observed. Although a reduction in reflectivity was observed from Film-A to Film-C throughout the entire wavelength range (Fig. 6(a)), a significant change in EQE was noticed only in the shorter wavelength region, Fig. 7(a). The reason for this observation could be ascribed to the change in band structure around the FSF layer, as well as its RI. An increase in the bending of the valence band (VB), reduces the hole collection or leakage to the FSF layer, the holes are increasingly being prevented to enter into the FSF layer of Cell-B than that in Cell-A, as demonstrated in Fig. 7(b). This band bending can also lead to field induced passivation. However, in Cell-C, and Cell-D, the reverse situation might have initiated; an increased band bending near the conduction band (CB) while at the VB the discontinuity is reduced. This situation can lead to an increased recombination at the FSF layer in these cells, thereby reducing the device performance. However, as Cell-C exhibits the lowest reflection, its EQE was the highest. Therefore, although an increased in the amount of light available to the absorber layer creates a larger number of electron-hole pairs, yet these excess carriers may not be collected at the front electrode due to disadvantageous band bending. This is especially true for Cell-C, and Cell-D. It is interesting to note that the silicon oxide layer can exhibit surface passivation to some extent^1^, therefore, an optimized n-µc-SiO:H layer can reduce the recombination loss of carriers. It is also possible that the difference in the band-gap of a-Si:H and FSF layers will be helpful in carrier selection, as shown in Fig. 7(b).

**Figure 8:**
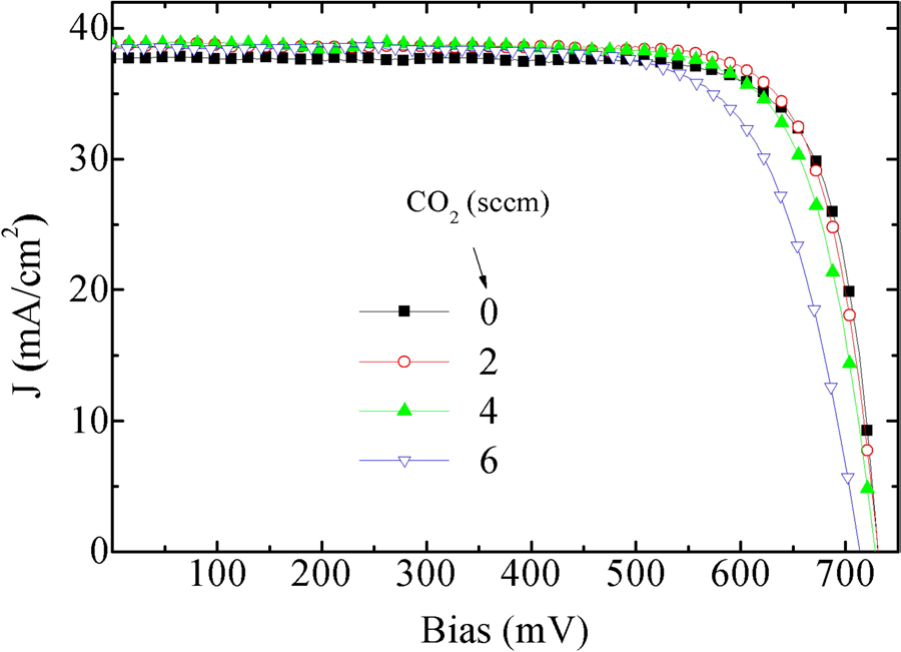
J-V characteristic curves of the fabricated cells, measured at AM1.5 G illumination and room temperature.

The device performance was quantitatively estimated from the current density-voltage characteristic curves, as shown in Fig. 8. The variation in device parameters can be better understood from Table 2. It shows that J_sc_ increases from Cell-A to Cell-C, but a decrease can be observed in Cell-D. Comparing to the optical reflectivity in Fig. 6, this corresponds to the amount of light that enters the cell. On the other hand, V_oc_ and PCE remain maximum for Cell-B and then decreased to Cell-D. Additionally, the FF remained highest for Cell-A, as electrical conductivity of Film-A (or the corresponding FSF layer) was the highest. Therefore, the highest observed efficiency was observed for Cell-B, with a J_sc_ of 38.71 mA/cm^2^ and FF of 78.95%. The PCE reported here is the maximum power conversion efficiency obtained from the J-V curves. Therefore, it seems reasonable to show the variation in output voltage (PmaxV) and current density (PmaxJ) at the maximum power point as shown in the last two columns in Table 2. Here it can be observed that PmaxV continuously decreased from Cell-A to Cell-D, while the PmaxJ was highest for Cell-B. Incidentally, our results are closely similar to that recently reported one, Cell C of ref.^15^ however our device exhibited a higher V_oc_ and J_sc_ (731.06 mV and 38.71 mA/cm^2^ respectively), even though the thickness of our absorber layer was higher (200 μm) than that of the corresponding layer in the above-cited reference (140 μm); the referenced cell exhibited V_oc_, J_sc_ and PCE as 729.5 mV and 38.4 mA/cm^2^, 22.4% respectively (see Cell C in ref.^15^). Furthermore, in Cell C of ref.^15^, a 10 nm thick nano-crystalline FSF layer was used. A thinner FSF layer probably contributed to the higher FF (as compared to our results) as reported in the ref.^15^.

**Table 2:** Solar cell parameters obtained from the characteristic J-V curves, shown in Fig. 8.

Cell number	CO_2_ (sccm)	V_oc_ (mV) (±1.0)	J_sc_ (mA/cm^2^) (±0.2)	FF (%) (±1.02)	PCE (%) (±0.51)	PmaxV (mV)	PmaxJ (mA/cm^2^)
Cell-A	0	730.75	37.67	79.36	21.84	622	35.12
Cell-B	2	731.06	38.71	78.95	22.34	614	36.39
Cell-C	4	728.20	38.84	76.54	21.65	606	35.72
Cell-D	6	713.85	38.51	73.26	20.14	566	35.58

Average error in device parameters, estimated from 5 samples of each type, are shown in each column, while PmaxV and PmaxJ were estimated from single curves in Fig. 8.

It seems that because of the variation in the FSF layer, there will be a change in the energy band structure, as schematically shown in Fig. 7(b). In ref.^15^, varying the contact layer induced a change in the blue response of the EQE spectra, which is, to some extent, similar to the change observed in our EQE spectra. This change in device characteristics indicates a change in the recombination of carriers as well as in the series resistance (R_s_) of the cell. An increased carrier recombination is thought to be related to an increased reverse saturation current density (J_0_)^40^. If photo-generated carriers are not collected efficiently, they can influence electric field distribution within the device, because of which the effective diode ideality factor (DIF) of the device can also increase. Therefore, DIF can also be called as a shielding parameter related to the electric field inside the device^43^; information on such parameters can be obtained by an analysis of the single diode-equivalent-circuit of the solar cells^39,40^. The calculated parameters are listed in Table 3.

**Table 3:** Extracted DC diode parameters of the solar cells obtained from the I-V curves, by using $J={J}_{sc0}-{J}_{0}[{e}^{[(qV+qJ{R}_{s})/(DIF.kT)]}]-(V+J{R}_{s})/{R}_{p},$ where ${J}_{sc0}$ is the strength of the current source in an equivalent single-diode model circuit, and k is the Boltzmann constant.

Cell number	CO_2_ (sccm)	DIF	J_0_ (A/cm^2^)	R_s_ (Ohm.cm^2^)	R_p_ (Ohm.cm^2^)
Cell-A	0	1.550	4.04 × 10^−10^	1.8 × 10^−15^	7442
Cell-B	2	1.480	1.79 × 10^−10^	0.20	3447
Cell-C	4	1.755	3.74 × 10^−9^	0.22	3692
Cell-D	6	2.115	7.94 × 10^−8^	0.24	6431

Table 3 shows the diode parameters of the solar cell under AM1.5 G insolation; these parameters were obtained from the I-V curves of Fig. 8, and by using numerical method. It is to be noted that sometimes R_s_ and R_p_ are estimated taking a slope at the open circuit and short circuit conditions, which can be called dynamic or AC resistance. In Table 3 we report the DC resistance values, as they exist in the diode equation. Table 3 shows that DIF, and the reverse saturation current density, J_0_, was the lowest for the Cell-B. This might be due to an increase in the optical band-gap from Film-A to Film-B, owing to which the bending of the VB around the FSF/a-Si:H interface increases favorably for higher rate of collection of photo-generated electrons and lower collection of holes. Such a change in carrier collection, in combination with the reduction in light reflection by the FSF layer leads to an improved J_sc_ in Cell-B. Figure 7(b) indicates that the valence band discontinuity increases in Cell-B, raising carrier selectivity due to the Film-B. An increased collection of carriers at the FSF/a-Si:H interface is also indicated by the reduction in J_0_ in Cell-B than that in Cell-A. However, R_s_ was found to increase from near zero in Cell-A to 0.20 Ohm.cm2 in Cell-B. This Rs can be termed as contact resistivity. This increase in R_s_ could be ascribed to the reduction in the dark conductivity of Film-B than that in Film-A, or to an increase in the resistivity of the FSF layer in Cell-B. An increase in R_s_ can be a reason for the reduction in FF that we see in all the cells from Cell-A to Cell-D, as shown in Table 2. The series resistances of the subsequent cells continue to increase, probably because of a continuous reduction in the electrical conductivity of the FSF layers (from Film-A to Film-D, as shown in Fig. 4(a)). On the other hand, the increase in the shunt resistance from Cell-B to Cell-D is favorable for device performance, whereas current shunting is disadvantageous. Therefore, a reduction in current shunting from Cell-B to Cell-D is another advantage of using FSF layers with wider optical band-gap. However, because the PCE are low for these cells, the overall effect of wider optical gap FSF layer does not induce a better performance than that observed in the optimum cell, Cell-B. It is expected that further developments is possible in future thereby it can lead to a further improvement in device efficiency.

Therefore, overall benefit in using the optimized nano-crystalline silicon oxide is that it seems to help in carrier selectivity and improvement in field induced passivation^1,5^, at the same time with an increased light transmission to the absorber layer. As a result, a lower carrier recombination is expected near the FSF layer. The silicon oxide layer has lower extinction coefficient, as indicated in Fig. 3(a), higher optical band-gap, as shown in Fig. 3(b). Therefore, the parasitic optical absorption in the silicon oxide based FSF layer will be lower than that in micro-crystalline silicon layer.

## Conclusions

We investigated n-µc-SiO:H for an optimum FSF layer in rear emitter heterojunction silicon solar cells. The opto-electronic properties of the n-µc-SiO:H layer influences the device performance. As n-µc-SiO:H layers can be prepared with varying optical band-gaps, refractive indices, electrical conductivities, crystallite volume fractions, it is possible to obtain an optimized material for a particular device structure. The refractive indices of the ITO layer and the a-Si:H passivation layers are different from each other. In order to reduce optical reflection at the interfaces of the thin films, the FSF layer that is sandwiched between these two layers, should have an intermediate refractive index. A reduced optical reflection can enhance the intensity of light available to the absorber layer. However, in order to enhance device efficiency, the large number of electron-hole pairs generated in the absorber layer, should also be collected efficiently. It seems that the collection efficiency of such carriers decreased due to a reduced electrical conductivity and mismatch in the energy band structure of the FSF layers. Therefore, neither the highest electrical conductivity (as obtained with 0 sccm CO_2_), nor the lowest optical reflection (as obtained with 4 sccm CO_2_) results in the best device performance. In our investigation, we concluded that an intermediate between the two conditions (2 sccm CO_2_ flow rate, or Film-B), with which best device performance was observed. This was Film-B and therefore Cell-B, having the optimum opto-electronic properties.
